# Doing Vulnerability: Soziale Konstruktionen des Alter(n)s während der COVID-19 Pandemie

**DOI:** 10.1007/s00391-022-02143-2

**Published:** 2022-12-20

**Authors:** Vera Gallistl, Karoline Bohrn, Rebekka Rohner, Franz Kolland

**Affiliations:** Kompetenzzentrum für Gerontologie und Gesundheitsforschung, Karl Landsteiner Privatuniversität für Gesundheitswissenschaften, Dr.-Karl-Dorrek-Straße 30, 3500 Krems, Österreich

**Keywords:** Doing Age, Ageism, Risikogruppe, Qualitative Studie, Kritische Gerontologie, Doing age, Ageism, Risk group, Qualitative research, Critical gerontology

## Abstract

**Hintergrund:**

Ein häufig wiederholtes Forschungsergebnis zu COVID-19 ist, dass ältere Menschen besonders vulnerabel gegenüber einem schweren Infektionsverlauf sind. Einerseits wurde, basierend darauf, für Altersdifferenzierungen in den COVID-19-Schutzmaßnahmen argumentiert, andererseits kritisierten Gerontolog*innen altersbasierte Risikogruppendefinitionen und einen ihnen zugrunde liegenden Ageismus.

**Ziel der Arbeit:**

Dieser Beitrag fragt danach, wie sich die Verwirklichung eines vulnerablen Alter(n)s im Alltag älterer Menschen während der COVID-19 Pandemie praktisch vollzogen hat.

**Material und Methoden:**

Der Beitrag bezieht sich auf Daten von 6 themenzentrierten Interviews mit Personen zwischen 65 und 87 Jahren. Die Daten wurden mithilfe einer Feinstruktur- und Themenanalyse ausgewertet.

**Ergebnisse:**

Die Ergebnisse verdeutlichen, dass Doing Age während der COVID-19-Pandemie durch ein Not Doing, ein Verschwinden von routinisierten Alltagspraktiken, gekennzeichnet war. Durch dieses Not Doing Age aktualisiert sich für die Befragten ein vulnerables Älterwerden, durch das sie körperliche und zeitliche Vulnerabilität intensiver erleben.

**Diskussion:**

Die praktische Verwirklichung des Alter(n)s während der COVID-19-Pandemie hat sich vor dem Hintergrund gesellschaftlicher Verhältnisse vollzogen; diese haben die Aktivität als einen Indikator für gutes und lebenswertes Altern festgelegt. Gleichzeitig wurden nur wenige Möglichkeiten geschaffen, Aktivität unter den Bedingungen der COVID-19-Pandemie aufrechtzuerhalten.

## Abstract (en)

**Background:**

Since the beginning of the COVID-19 pandemic old age has been “vulnerablilized” in the media by characterizing old age as a vulnerable and risky phase of life. Gerontologists have criticized the age-based definition of risk groups for their underlying agism.

**Objective:**

From a practice-theoretical perspective the article asks through which social practices a vulnerable age is realized during the COVID-19 pandemic.

**Material and methods:**

The article refers to data from six qualitative interviews with people aged 65 and older. The data was analyzed using fine structure analysis followed by a topic analysis.

**Results:**

The results show that during the COVID-19 pandemic doing age was characterized by a not doing or the disappearance of routinized everyday practices. For the respondents this not doing age was connected to experiencing vulnerable ageing, in which they felt physically and temporally vulnerable.

**Discussion:**

Practices of aging during the COVID-19 pandemic have developed against the backdrop of a societal discourse that has positioned activity as an indicator of good and positive aging. At the same time few opportunities have been created to maintain activity under the conditions of the COVID-19 pandemic.

## Hintergrund und Fragestellung

Ein häufig wiederholtes Forschungsergebnis zu COVID-19 ist, dass ältere Menschen überdurchschnittlich häufig von einem schweren Infektionsverlauf betroffen und überproportional unter jenen Personen vertreten sind, die mit einer Infektion versterben [17, 28]. Diese Evidenz wurde im Verlauf der Pandemie als Basis zweier Argumentationslinien herangezogen, die die soziale Konstruktion des Alter(n)s während der COVID-19-Pandemie kennzeichnen. Einerseits wurde für Altersdifferenzierungen in den COVID-19-Schutzmaßnahmen argumentiert; alte Menschen wurden als Risikogruppe [4] adressiert und das Alter damit deutlich vulnerabilisiert [13]. Andererseits kritisierten Gerontolog*innen altersbasierte Risikogruppendefinitionen aufgrund eines ihnen zugrunde liegenden Ageismus [7, 24] und positionierten sich als diskursives Gegengewicht zur medialen Debatte. Was diese beiden Positionen eint, ist ein Blick auf das Alter als eine „biosoziale Achse“ [8, S. 484] sozialer Differenzierung in der COVID-19-Pandemie; dieser hat das Alter als chronologisches Merkmal sozialstruktureller Differenzierung von Vulnerabilität (wieder) ins gesellschaftliche Bewusstsein geholt.

Der vorliegende Beitrag untersucht diese Konstruktionen des Alter(n)s während der COVID-19-Pandemie auf der Ebene sozialer Praktiken (*Doing Age*, [26]). Er fragt danach, wie sich die Verwirklichung eines vulnerablen Alter(n)s im Alltag älterer Menschen während der COVID-19-Pandemie praktisch vollzogen hat. Im Sinne einer kritischen Gerontologie geht es dabei nicht um die normative Ablehnung von negativen Alterskonstruktionen. Eher zielt der Artikel darauf ab zu fragen, vor dem Hintergrund welcher gesellschaftlicher Machtverhältnisse sich Alterskonstruktionen vollziehen [20], sowie die „interpretierenden Bedeutungsmuster“ [27, S. 23] und soziale Praktiken sichtbar zu machen, durch die Alterskonstruktionen ihre gesellschaftliche Wirkung entfalten.

## Doing Vulnerability und kritische Gerontologie

Wie lässt sich aus einer kritisch-gerontologischen Perspektive Vulnerabilität im Alter verstehen? Ursprünglich aus den Umweltwissenschaften kommend wird das Konzept der Vulnerabilität in der Gerontologie verwendet, um das Ausgesetztsein („exposure“) eines älteren Individuums gegenüber einer potenziellen Gefährdung („threat“) sowie die individuelle Fähigkeit, auf diese Gefahr zu reagieren, („coping capacity“) zu beschreiben [25]. Der Begriff der Vulnerabilität beschreibt eine erhöhte individuelle „Verletzlichkeit“ oder „Empfänglichkeit“ [21, S. 460] für chronische Erkrankungen oder funktionelle Einschränkungen, die mit dem höheren Alter einhergehen. Dieser „Verletzlichkeitsperspektive“ [21, S. 459] müsse allerdings – so Kruse – eine „Potenzialperspektive“ [21], die die Entwicklungspotenziale älterer Menschen betont, zur Seite gestellt werden.

Gemeinsam ist diesen beiden Perspektiven zur Vulnerabilität im Alter der Fokus auf das ältere Individuum, dessen Vulnerabilität sich aus der biologischen Alterung, der individuelle Coping-Strategien „gegengerechnet“ werden können, quasi natürlich ergibt [11]. Ein solcher „restriktiver“ [3] Begriff von Vulnerabilität, der das Entstehen von Verletzlichkeiten in bestimmten individuellen Lebenssituationen – etwa dem höheren Lebensalter – verortet, bestimmte den öffentlichen Diskurs und die gerontologische Replik zu älteren Risikogruppen von COVID-19.

Es stellt ein Charakteristikum der kritischen Gerontologie dar, die Gesellschaft als konstitutiv für Aspekte des Alter(n)s in den Blick zu nehmen [2]. Ein kritisch-gerontologischer Begriff der praktischen *Vulnerabilisierung* („doing vulnerability“), der hier angelegt wird und der über den der (individuellen) Vulnerabilität hinausgeht, verweist auf die politischen, sozialen und gesellschaftlichen Machtmechanismen, die bestimmte Personengruppen oder Lebensphasen als vulnerabel kennzeichnen [13]. Hintergrund dafür ist ein breiteres Konzept von Vulnerabilität, das auf die grundsätzliche Verbundenheit von menschlichen Körpern mit den sozialen und materiellen Relationen einer Gesellschaft hinweist [5] und Vulnerabilität versteht, als eine umfassende, ontologische Bedingung menschlicher Existenz [3], die durch gesellschaftliche Prozesse sozial ungleich verteilt sichtbar, erlebbar und problematisiert wird [12]. Wird ein solches Verständnis der Vulnerabilität des Menschen vorausgesetzt, so lässt sich danach fragen, warum und unter welchen gesellschaftlichen, politischen und materiellen Bedingungen die Vulnerabilität von hochaltrigen oder pflegebedürftigen Menschen während der COVID-19-Pandemie stärker sichtbar gemacht wurde als von anderen Gruppen. Angesprochen ist damit auch die gesellschaftlich ungleich verteilte Akzeptanz von Vulnerabilität: Wessen Vulnerabilität wird gesellschaftlich hingenommen, akzeptiert und normalisiert? Welche Vulnerabilität wird als gesellschaftlicher Handlungsauftrag für Intervention oder verstärkte Sorge verstanden^1^?

## Studiendesign und Untersuchungsmethoden

Es werden Ergebnisse einer qualitativen Studie vorgestellt, in der Interviewmaterial zur Alltagsgestaltung älterer Menschen während der COVID-19-Pandemie erhoben wurde. Es wurden themenzentrierte Interviews zwischen September und Oktober 2021 mit 6 Personen (3 Frauen, 3 Männer) zwischen 65 und 87 Jahren geführt (durchschnittliche Interviewdauer 76 min). Das Interviewmaterial wurde zunächst mithilfe einer Feinstrukturanalyse von zentralen Interviewpassagen sowie der Einstiegserzählung der Interviews analysiert [22]. Danach wurden themenanalytisch die zentralen Themen der Interviews aufgearbeitet und zwischen den Interviews verglichen [9]. Alle Interviewzitate wurden für den Zweck dieses Artikels anonymisiert und von den Autor*innen in die deutsche Standardsprache übersetzt.

## Ergebnisse

Die Interviews zeigen, dass Narrative zur COVID-19-Pandemie eng mit Narrativen des Alter(n)s verknüpft sind. Gefragt nach ihren Erfahrungen mit der Pandemie sprechen die Befragten die Kategorien „alt“ und „älter“ an, um ihr subjektives Erleben der Pandemie zu beschreiben, wobei etwa für Frau Gruber deutlich wird: „Älter habe ich mich nicht gefühlt, aber ich habe mich alt gefühlt“ (S. 25, Z. 3–4). Einen möglichen Kontext für dieses subjektive Erleben des Altseins führt Frau Winkler in ihrem Interview aus. Sie erklärt, dass sie eine mediale und politische Adressierung älterer Menschen als Risikogruppe wahrgenommen habe, in der Älteren Hilfslosigkeit zugeschrieben wurde: „Es wurden die Alten nur als vollkommen hilflose Wesen dargestellt, finde ich. Was ja eigentlich im Prinzip nicht stimmt. … Weil, wenn ich denk’, wie viele Alte, weiß ich wohin, reisen und, was weiß ich, was unternehmen, aber man wollte das Mitleid heraufbringen quasi“ (S. 14, Z. 5–8).

Frau Winkler zieht zwei konkrete Praktiken (reisen und etwas unternehmen) als Argument heran, warum ältere Menschen nicht pauschal hilflos sind. Hilflos sind für Frau Winkler jene älteren Menschen, die keiner dieser oder ähnlicher Aktivitäten nachgehen wollen oder können. Der Geschäftigkeitsethos des Alter(n)s [18], der ein hohes Aktivitätsniveau im Alter mit gutem und selbstständigem – im Beispiel von Frau Winkler: nichthilflosem – Alter gleichsetzt, zeigt sich damit auch in den Alterskonstruktionen der COVID-19-Pandemie.

### Doing Vulnerability und Not Doing Age

Der Alltag in Zeiten der COVID-19-Pandemie war für die Befragten dadurch gekennzeichnet, bestimmte Dinge aufgrund der COVID-19-Schutzmaßnahmen nicht mehr tun zu können (*Not Doing*). Für Frau Winkler, die ihren Sportkursen nicht mehr länger nachgehen konnte, aktualisiert sich damit in ihrem Alltag altersbezogene Vulnerabilität, weil sie so körperliche Einschränkungen intensiver erlebt: „Es kommt einem dann viel mehr zu Bewusstsein, dass es körperlich nicht mehr so gut geht. Es hat auch damit zu tun, dass all die Sachen, die Bewegung usw. so gebraucht haben, nicht stattgefunden haben. Und damit wird alles noch mühsamer, weil das dann nicht mehr so geht. Das ist halt auch so, dass ich sage, das werde ich wahrscheinlich nicht mehr aufholen. Ich meine, für die Jungen kein Problem“ (S. 5, Z. 22–27). Der Aktivitätsverlust durch das Wegfallen von Bewegungsprogrammen wurde von Frau Winkler als Praxis der Vulnerabilisierung erlebt, weil ihr dadurch ihre körperliche Verletzlichkeit ins Bewusstsein gebracht wurde.

Neben dieser körperlichen Vulnerabilisierung wurde in den Interviews auch eine zeitliche Vulnerabilisierung beschrieben – und zwar dort, wo die Befragten beschreiben, wertvolle Lebenszeit „verloren“ zu haben, die sie im Alter als rar betrachten. So beschreibt Frau Weiß: „Allgemein, dass ich sehr bedaure, dass wir irgendwo die letzten oder die eineinhalb letzten Lebensjahre irgendwo verloren haben. … Wir sind immer ins Konzert gegangen, ins Theater gegangen, wir haben drei Abonnements gehabt, und mit einem Schlag war das alles aus“ (S. 6, Z. 1–5). Die Vulnerabilisierung des Alter(n)s hat sich also nicht nur auf einer körperlichen, sondern – für Frau Weiß – auch auf einer kulturellen, zeitlichen Ebene verwirklicht, da ihr die Begrenztheit des Lebens vor Augen geführt wurde.

## Diskussion

Die Ergebnisse verdeutlichen, dass das *Doing Age* während der COVID-19-Pandemie durch ein *Not Doing*, einen Verlust an routinisierten Praxiskonstellationen (wie Theater- und Konzertbesuche oder Bewegungsangebote) gekennzeichnet war. Durch dieses *Not Doing Age* aktualisiert sich für die Befragten ein *vulnerables Älterwerden*, in dem sie sich alt (Frau Gruber) oder körperlich angeschlagen (Frau Winkler) fühlen, oder sich stärker Gedanken über die zeitliche Begrenztheit ihres Lebens machen (Frau Weiß). Dabei wird auch deutlich, dass sich die praktische Verwirklichung des Alter(n)s während COVID-19 vor dem Hintergrund gesellschaftlicher Verhältnisse, die ältere Menschen in den letzten Jahrzehnten als produktive Ressource adressiert und Aktivität als einen Indikator für gutes, produktives und lebenswertes Altern festgelegt haben, vollzieht [10]. Gleichzeitig wurden in der Pandemie wenige Möglichkeiten für ältere Menschen geschaffen, diese Aktivität auch unter den Bedingungen von Social Distancing aufrechtzuerhalten. Das kulturelle Versprechen eines aktivierenden Staates [1], dass mit erhöhter Aktivität und Produktivität der Älteren für die Gesellschaft auch mit ein höheres Maß an sozialer Teilhabe und Partizipation einhergeht, wurde im Verlauf der COVID-19-Pandemie auf zwei Ebenen gebrochen: Einerseits kam es nur zu wenig Mitbestimmung älterer Menschen über jene politischen Maßnahmen, die sie als Risikogruppe adressierten; andererseits wurde in jenen Bereichen, in denen ältere Menschen jenseits des Erwerbslebens soziale Teilhabe erleben (ehrenamtliche Tätigkeiten, nachberufliche Bildung, kulturelle Veranstaltungen), die Teilhabe durch COVID-19-Schutzmaßnahmen erschwert.

Für die Konzeptionalisierung von *Doing Age* [26] zeigen die Ergebnisse auf, dass das Alter(n) auch durch das Wegfallen und Unterlassen von Aktivitäten hergestellt wird. Ein *Not Doing Age*, wie es hier vorgeschlagen wird, meint damit nicht die Negation von Altersdifferenzen im Sinne eines *Undoing Age* [14, 16], sondern soll dafür sensibilisieren, dass das *Doing Age* durch unterschiedliche Aktivitätsniveaus, und dadurch nicht nur ein aktives Tun, sondern auch durch das Unterlassen, Aufgeben oder Einstellen von Aktivitäten praktisch hergestellt wird [15].

Für die kritische Gerontologie unterstreichen die Ergebnisse die Notwendigkeit, die Frage danach zu stellen, wie selbstbestimmtes Altern unter den Bedingungen breiter gesellschaftlicher Vulnerabilisierung, verbunden mit dem Geschäftigkeitsethos eines aktivierenden Wohlfahrtsstaats, gelingen kann. So geht es wohl nicht um die Ablehnung von Vulnerabilität im Alter, sondern darum, gesellschaftliche Anreize zu schaffen, damit dieser nicht mit Exklusion und Rückzug, sondern (Für- oder Selbst‑)Sorge begegnet werden kann. Diese Anreize in Form von alternativen sozialen Teilhabemöglichkeiten (z. B. digital) haben in der COVID-19-Pandemie weitgehend gefehlt. Dabei geht es nicht nur darum, ältere Menschen zum Ausgangspunkt gesellschaftlicher Sorge zu machen (sich Sorgen um), sondern auch darum, mit Vulnerabilität auf eine ethisch verträgliche Art und Weise umzugehen (Sorge tragen für) und Aktivität jenseits von einem Geschäftigkeitsethos des Alter(n)s zu ermöglichen.

Welche Impulse lassen sich für die Veränderung der gesellschaftlichen Verhältnisse, die das Alter(n) als vulnerabel konstituieren, formulieren? Erstens geht es darum, die medialen Erzählungen zum Alter hin zu einer differenzierten Sichtweise, die die Heterogenität älterer Menschen, sowie deren Impulse zur Selbstvertretung und Aktivitäten der Selbstsorge in den Vordergrund stellt, zu fördern und damit einen Blick auf das ambivalente Erleben von Vulnerabilität im Alter zu ermöglichen. Studien haben gezeigt, dass sich ältere Menschen selbstbestimmt Alternativen zu Aktivitäten während der Pandemie gesucht haben [23] und sich der eigenen Vulnerabilität selbstsorgend zuwenden [19]. Zweitens geht es um die Unterstützung von zivilgesellschaftlichem Engagement älterer Menschen als Aktivitäten der (Selbst‑)Sorge zur Bearbeitung von Vulnerabilität und den Aufbau von Gelegenheitsstrukturen, um gesellschaftliche Sorgepraktiken für und mit älteren Menschen möglich zu machen.

Relevante Limitationen dieser Studie sind, dass Daten in Niederösterreich und Wien erhoben wurden und sich die Analyse auf eine kleine Stichprobe von 6 Personen bezieht. Zusätzlich handelt es sich um Interviewpartner*innen, die eine hohe Bildung und gute finanzielle Ressourcen aufweisen, was primär die Sichtweisen einer gut situierten Mittel- bis Oberschicht darstellt. Soziale Ungleichheiten konnten mit den vorliegenden Daten deshalb wenig analysiert werden.

## Fazit für die Praxis

- Das kulturelle Versprechen, dass mit erhöhter Aktivität und Produktivität der Älteren für die Gesellschaft ein höheres Maß an sozialer Teilhabe und Partizipation einhergeht, wurde im Verlauf der COVID-19-Pandemie gebrochen: Ältere Menschen konnten über politische, Maßnahmen, die sie als Risikogruppe adressierten, nur wenig mitbestimmen, gleichzeitig wurde der Zugang zu jenen Bereichen, in denen sie soziale Teilhabe jenseits des Erwerbslebens erleben könnten (z. B. ehrenamtliches Engagement), durch Schutzmaßnahmen erschwert.
- Vorgeschlagen wird die Konzeption einesvon Not Doing Age, das dafür sensibilisieren soll, dass Doing Age neben aktivem Tun auch durch das Unterlassen, Aufgeben oder Einstellen von Aktivitäten hergestellt wird.
- Der Beitrag stellt die Frage, wie selbstbestimmtes Altern unter den Bedingungen gesellschaftlicher Vulnerabilisierung gelingen kann. Es müssen Anreize und entsprechende Unterstützung schaffen werden, damit dieser nicht mit Exklusion und Rückzug, sondern (Für- oder Selbst‑)Sorge begegnet werden kann. Gelegenheitsstrukturen, die gesellschaftliche Sorgepraktiken für und mit älteren Menschen ermöglichen, müssen aufgebaut werden.

## Footnotes

1 Im Weiteren gerät auch die sozial ungleich verteilte „grievability“ (Fähigkeit zu trauern) in den Blick: Wessen Tod wird gesellschaftlich hingenommen, und wessen als gesellschaftlicher Handlungsauftrag für die Zukunft verstanden [6]?
